# Reasons for choosing to specialise in psychiatry: differences between core psychiatry trainees and consultant psychiatrists

**DOI:** 10.1192/pb.bp.114.048678

**Published:** 2016-02

**Authors:** Melissa Denman, Femi Oyebode, Jayne Greening

**Affiliations:** 1 University of Birmingham; 2 Birmingham and Solihull Mental Health NHS Foundation Trust

## Abstract

**Aims and method** This questionnaire study aimed to investigate the reasons for choosing to specialise in psychiatry in a sample of consultant psychiatrists and core trainee psychiatrists from within the West Midlands.

**Results** Five reasons were significantly different between the core trainees and consultant psychiatrists. ‘Emphasis on the patient as a whole’ was identified as the most important reason for choosing to specialise for both core trainees and consultants. Six additional reasons were shared within the top ten ‘very important’ reasons, although their actual ranking varies.

**Clinical implications** Some of the reasons for choosing to specialise in psychiatry were shown to significantly differ between core trainees and consultants. Numerous key driving factors have remained important over time for both groups, whereas other reasons have been replaced with a shift of importance towards lifestyle and humanitarian factors for core trainees. Consequently, it may be advisable not to use the reasons that consultants gave for choosing psychiatry when thinking about how to attract today's prospective psychiatrists.

The recruitment and retention of psychiatrists is a long-standing concern. The Royal College of Psychiatrists' annual census in 2013 revealed that 5.9% of psychiatry consultant posts in England were unfilled, with a further 14.8% filled by locums.^1^ Census figures demonstrate a dramatic and unsustainable 93.9% increase in the number of vacant and unfilled consultant posts since 2011.^2^ In response to the long-standing shortage of psychiatrists, the College developed a 5-year recruitment strategy.^3^ To increase recruitment it is essential to identify the ‘pull factors’ which may then be used to attract prospective psychiatrists into the specialty. Numerous studies have considered factors associated with choosing to specialise in psychiatry – the majority have been carried out on prospective medical students,^4^ current medical students^5-7^ and medical graduates.^8^ Interestingly, studies carried out on practising psychiatrists remain few. A systematic literature search performed on Ovid EMBASE database (using the following combination of keywords: psychiatry/or psychiatr* AND career/or career planning AND reasons) produced 21 results, of which 3 were identified as relevant. Two further papers were identified from scanning of references. Of these 5 papers, only 2 studies were conducted in the UK.^9,10^ This means that researchers have been neglecting to utilise the strong pull factors identified within the population who dedicated their careers to psychiatry. Moreover, both these studies collected data from and reported solely on consultant psychiatrists who on average had 21 years of experience in psychiatry.^9^ Considering the rapid advances in the practice of medicine and the changes in the employment environment it is questionable whether these factors, influential 21 years ago, are relevant to today's graduates.

A study comparing reasons for specialising in psychiatry between core trainees and consultant psychiatrists is required to better inform researchers on the relevance of using consultant psychiatrists in such studies and to identify current motives for choosing to specialise in psychiatry to aid future recruitment strategies.

## Method

A structured questionnaire was designed based on a model used in a previous study by Dein *et al*^9^ (the questionnaire is available from the authors on request). The questionnaire has two sections: general participant information and factors influencing career choice. Forty-five factors associated with choosing to specialise in psychiatry were identified from a systematic review of the literature. A group interview was conducted with seven practising psychiatrists to ensure that as large a range of reasons as possible had been captured and this resulted in the addition of three more factors. The final 48 factors were included in section 2 (under specific headings: job content factors; lifestyle factors; personal factors; experience factors), where participants were instructed to indicate on a 4-point Likert scale how important they perceived each factor to be in influencing their decision to specialise in psychiatry. The questionnaire was then piloted, which did not result in any further changes. All consultant psychiatrists from Birmingham and Solihull Mental Health Foundation Trust (BSMHFT) and all core trainees (CT1–CT3) from the West Midlands School of Psychiatry received an email invitation to complete the online questionnaire. A weekly reminder email was sent over the course of 3 weeks. Additionally, participants were recruited at core trainee training sessions and consultant meetings within the Trust and completed paper questionnaires.

### Analysis

Data were analysed using descriptive statistics and SPSS 21 for Windows. The 48 factors influencing career choice were each analysed individually. The data were non-normally distributed therefore medians were reported and a series of Mann–Whitney *U*-tests were performed to test for a difference between the core trainees' and consultant psychiatrists' responses. The median for each factor was calculated for core trainees and consultant psychiatrists separately. *P*-values were adjusted by Bonferroni correction to reduce the chances of type I errors due to multiple testing.^11^

## Results

### Participants

Forty-seven (42%) consultants out of 112 employed in BSMHFT in March 2014 responded to the invitation to complete the questionnaire. Of the 125 West Midlands core trainees in March 2014, 51 (41%) responded. The participants' demographic characteristics are summarised in Table 1.

**Table 1:** Demographic characteristics of core psychiatry trainees and consultant psychiatrists

	Core trainees (*n* = 51)	Consultants (*n* = 47)
	
	*n* (%)	
Female	33 (65)	21 (45)

Male	18 (35)	26 (55)

Full-time	41 (80)	39 (83)

Part-time	10 (20)	7 (15)

Semi-retired	0 (0)	1 (2)

With regard to the consultant population, years of experience working as a psychiatrist ranged from 7 to 36 (median 20 years) starting from CT1 level or equivalent. The most common subspecialty was general adult psychiatry (53%), followed by old age psychiatry (17%), forensic psychiatry (15%), intellectual disability psychiatry, child and adolescent psychiatry and psychotherapy (4% each) and neuropsychiatry (2%).

### Stage of choosing psychiatry

**Fig. 1:**
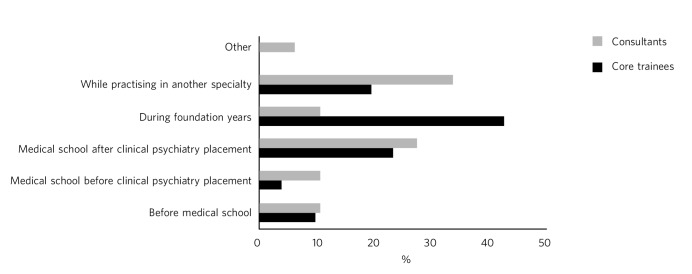
Stage of choosing to specialise in psychiatry.

The data are summarised in Fig 1. The timing of the decision to specialise in psychiatry differed markedly between the consultants and core trainees. Around 50% of consultants had already made the choice on graduation from medical school and an additional 34% moved into psychiatry from another specialty. However, the largest proportion of core trainees (*n* = 22, 43%) made their decision to specialise in psychiatry during their foundation years, with 37% of core trainees having made the decision to specialise on graduation from medical school.

### Country of primary medical qualification

Thirty consultants obtained their primary medical degree in the UK (64%) and 17 (36%) graduated overseas. Of the core trainees, 34 obtained their primary medical degree in the UK (67%) and 17 (33%) graduated overseas.

### Reasons for choosing to specialise in psychiatry

Five reasons associated with choosing to specialise in psychiatry were rated significantly higher (*P*<0.05) by core trainees than by consultants (Table 2; full table including statistics for all 48 factors is available from the authors on request). They were: the expectation that working hours in psychiatry are more compatible with family life (*u* = 732.50; *P*⩽0.001); better working conditions expected within psychiatry (*u* = 688.00; *P*⩽0.001); salary (*u* = 707.00; *P* = 0.048); psychiatry perceived to offer a better quality of life compared with other specialties (*u* = 758.50; *P* = 0.048); and opportunity to understand yourself better through working with patients (*u* = 702.00; *P*⩽0.001). The remaining 43 factors were not significantly different after correction for multiple testing.

**Table 2:** Significant factors associated with choosing to specialise in psychiatry in core psychiatry trainees and consultant psychiatrists

Factor	Core trainees (*n* = 51) Median^a^ (range)	Consultants (*n* = 47 ) Median^a^ (range)	*u*	*P*	*P^b^*
Lifestyle factors					
Working hours	3.41 (3)	2.65 (3)	732.50	<0.001	<0.001
Working conditions	3.23 (3)	2.43 (3)	688.00	<0.001	<0.001
Salary	2.32 (3)	1.66 (2)	707.00	0.001	0.048
Quality of life	3.21 (3)	2.35 (3)	758.50	0.001	0.048

Personal factors					
Self-understanding	3.00 (3)	2.19 (3)	702.00	<0.001	<0.001

a. Responses were scored on a 4-point Likert scale (1, unimportant to 4, very important).

b. After applying Bonferroni's correction.

#### The ‘very important’ reasons influencing the decision to specialise in psychiatry

The ten reasons ranked ‘very important’ in influencing the decision to choose psychiatry as a career for core trainees and consultants are shown in Table 3. The most common reason was the same for both groups: 28 (60%) core trainees and 33 (70%) consultants indicated that the emphasis on the patient as a whole person was very important in influencing their decision to specialise in psychiatry. Six additional factors were shared within the top ten highly rated factors between trainee and consultant psychiatrists.

**Table 3:** Top ten factors rated ‘very important’ in influencing the decision to specialise in psychiatry

	Core trainees (*n* = 51)	Consultants (*n* = 47)
	
Factors	*n* (%)	
Emphasis on the patient as a whole person	28 (60)	33 (70)

Empathy and concern for mentally ill people^a^	27 (53)	

Mental health considered an area of need^a^	27 (53)	

Expectation of working hours being more compatible with family life^a^	25 (49)	

Original and unique themes encountered	25 (49)	26 (55)

Importance of social and relational issues	23 (45)	24 (51)

Opportunity to form long-term doctor-patient relationships	22 (43)	18 (38)

Self-assessment of suitability for psychiatry	22 (43)	17 (37)

More interested in people than diseases	22 (43)	17 (36)

Importance of narratives and meaning more than of technology	21 (42)	24 (52)

Intellectually challenging^b^		28 (60)

Fulfilment when seeing patients improve^b^		22 (47)

Enjoyment of problem-solving^b^		22 (47)

a. Factors identified by core trainees only.

b. Factors identified by consultants only.

Factors within the top rated ‘very important’ reasons for core trainees but not for consultants were: empathy and concern for people with mental illness (*n* = 27, 53%); mental health considered an area of need (*n* = 27, 53%); and expectation of working hours in psychiatry being more compatible with family life (*n* = 25, 49%).

Conversely, factors within the top rated ‘very important’ reasons for consultants but not for core trainees were: that a career in psychiatry would be intellectually challenging (*n* = 28, 60%); sense of fulfilment expected from seeing patients improve (*n* = 22, 47%); and enjoyment of problem-solving (*n* = 22, 47%).

Participants were asked to indicate other reasons which influenced their decision to specialise in psychiatry, and these were: opportunity to make most impact to society as mental health problems are common and poorly understood (3 core trainees and 1 consultant); everyday variety (1 core trainee and 1 consultant); interest in psychology (2 core trainees); psychiatry professionals seem as amiable (1 core trainee, 1 consultant); easier to find employment (1 core trainee, 1 consultant); and an interest in spirituality and psychiatry (1 core trainee).

## Discussion

The response rate for this study was over 40% for both core trainees and consultant psychiatrists. The sample population (Table 1) was similar to the UK psychiatrist population in terms of gender and subspecialty when compared with the 2013 Royal College of Psychiatrists' census,^1^ and therefore it is likely to be representative of UK psychiatrists as a whole.

This study highlights a shift over the years concerning the stage at which the decision to choose to specialise in psychiatry is made. Over a third of consultants came into psychiatry from another specialty, whereas only a fifth of core trainees did so. The majority of core trainees made the decision to specialise during foundation years, which is likely to be a direct consequence of the increased exposure to psychiatry in foundation years. It is noteworthy that a large majority of core trainees in this study (*n* = 41, 80%) had a psychiatry job in foundation years. This opportunity was not available to most consultants. Kelley *et al*'s study^12^ found that foundation doctors exposed to psychiatry before specialty applications are nearly ten times more likely to choose to specialise in psychiatry. This adds weight to calls to grow the number of foundation psychiatry posts in a bid to increase recruitment. However, interestingly, the proportion of future psychiatrists choosing to specialise in psychiatry before medical school remains unchanged over time, perhaps reflecting the handful of individuals with an innate interest in psychological aspects of medicine who enter medical school with the intent to take up psychiatry.

The percentage of UK graduates entering psychiatry has remained consistent over time, confirming the extent to which international medical graduates continue to disguise the long-standing under-recruitment of UK graduates to psychiatry.

### Reasons for choosing to specialise in psychiatry

This study has demonstrated that 5 of the 48 factors associated with choosing to specialise in psychiatry significantly differ in importance between core trainees and consultant psychiatrists (Table 2). All of the five factors were scored as more important by the core trainee population than by the consultant population. It is of note that four of the five factors are lifestyle factors and relate to quality of life. One possible interpretation of these results is that working hours, salary and quality of life have become of increasing importance over the past 20 years. It may be that the simultaneous rise in the numbers of female psychiatrists over the years^13^ is responsible for the demonstrated differences in the importance of lifestyle factors. Additionally, perhaps a cultural shift in family roles has led to a growth in the number of men who consider work–family balance an important factor when choosing a career. Goldacre *et al*^14^ found that a higher percentage of graduates choosing psychiatry rated ‘domestic circumstances’ and ‘hours and working conditions’ as influential than did those choosing other careers.

Emphasis on the patient as a whole person was the top ‘very important’ rated factor for both core trainees and consultants. This is possibly what separates psychiatry from other medical specialties except general practice. Six of the top ten ‘very important’ factors were shared between the core trainees and consultants. They are likely to represent key drivers into psychiatry which have remained significant over the years. However, the rank in which the factors appear in the top ten has altered. There appears to be a greater vocational drive operating within the core trainees to work with the underprivileged, with empathy and concern for people with mental illness and mental health being considered an area of need ranking joint second. This drive is not apparent within the consultants' reasons for choosing to specialise in psychiatry, as their top ten ‘very important’ factors indicate more academic motives for a career within psychiatry by the inclusion of reasons such as a career in psychiatry being intellectually challenging and enjoyment of problem-solving.

### Limitations

This study was carried out among consultant psychiatrists and core trainees within a single area of the UK. A retrospective survey of this nature may lead to systematic recall bias which is likely to be greater for the consultant group than for the core trainees. Direct questioning may not always reveal true motivations behind career choice.^15^ Additionally, surveys are liable to socially desirable responses which may have affected the results of this study.

### Suggestions for future research

This study highlights a change in the stage of career in which doctors choose to embark on a career in psychiatry. It would be interesting to compare these results to those of core trainees in other specialties to investigate whether this finding indicates a global trend or is isolated and particular to psychiatry. Additionally, this study has identified a growing importance of lifestyle factors when choosing a career and it would be useful to discover whether this trend also appears across other specialties.

This study establishes that the reasons for choosing to specialise in psychiatry differ between core trainees and consultant psychiatrists. Numerous key driving factors for psychiatry have remained important over time, whereas other previously important factors have been displaced: academic factors seem to have been replaced by more domestic matters (work–life balance, salary and quality of life). The results of this study confirm that it is not appropriate to generalise the consultants' reasons for choosing to specialise in psychiatry to today's prospective psychiatrists. Today's recruitment strategies may be improved by promoting the likely quality of life benefits that choosing a career in psychiatry can offer in an attempt to boost psychiatry recruitment.
